# QPLOT Neurons—Converging on a Thermoregulatory Preoptic Neuronal Population

**DOI:** 10.3389/fnins.2021.665762

**Published:** 2021-05-04

**Authors:** Brian A. Upton, Shane P. D’Souza, Richard A. Lang

**Affiliations:** ^1^The Visual Systems Group, Abrahamson Pediatric Eye Institute, Cincinnati Children’s Hospital Medical Center, Cincinnati, OH, United States; ^2^Division of Pediatric Ophthalmology, Center for Chronobiology, Cincinnati Children’s Hospital Medical Center, Cincinnati, OH, United States; ^3^Molecular and Developmental Biology Graduate Program, College of Medicine, University of Cincinnati, Cincinnati, OH, United States; ^4^Medical Scientist Training Program, College of Medicine, University of Cincinnati, Cincinnati, OH, United States; ^5^Division of Developmental Biology, Cincinnati Children’s Hospital Medical Center, Cincinnati, OH, United States; ^6^Department of Ophthalmology, College of Medicine, University of Cincinnati, Cincinnati, OH, United States

**Keywords:** QRFP, PTGER3, Leptin receptor, Opn5, Tacr3, thermoregulation, torpor, neuropsin

## Abstract

The preoptic area of the hypothalamus is a homeostatic control center. The heterogeneous neurons in this nucleus function to regulate the sleep/wake cycle, reproduction, thirst and hydration, as well as thermogenesis and other metabolic responses. Several recent studies have analyzed preoptic neuronal populations and demonstrated neuronal subtype-specific roles in suppression of thermogenesis. These studies showed similar thermogenesis responses to chemogenetic modulation, and similar synaptic tracing patterns for neurons that were responsive to cold, to inflammatory stimuli, and to violet light. A reanalysis of single-cell/nucleus RNA-sequencing datasets of the preoptic nucleus indicate that these studies have converged on a common neuronal population that when activated, are sufficient to suppress thermogenesis. Expanding on a previous name for these neurons (Q neurons, which reflect their ability to promote quiescence and expression of *Qrfp*), we propose a new name: QPLOT neurons, to reflect numerous molecular markers of this population and to capture its broader roles in metabolic regulation. Here, we summarize previous findings on this population and present a unified description of QPLOT neurons, the excitatory preoptic neuronal population that integrate a variety of thermal, metabolic, hormonal and environmental stimuli in order to regulate metabolism and thermogenesis.

## Introduction

Body temperature regulation is key to survival and reproductive fitness with many mechanisms of regulation evolving throughout speciation. Obligate endotherms, such as mammals, use a variety of processes to regulate temperature, including the generation of heat via both shivering and non-shivering thermogenesis. Shivering thermogenesis results from rapid muscle contractions, whereas non-shivering thermogenesis takes place in specialized fat depots (brown adipose tissue; BAT) via uncoupling of mitochondrial gradients from the electron transport chain mediated by uncoupling protein 1 (Ucp1) (Nedergaard et al., 2001). This utilization of stored energy across the gradient is released as free energy that would have otherwise been incorporated into ATP. In addition to thermogenesis, organisms can conserve heat by seeking a warmer environment, and increasing blood flow to deep organs while decreasing cutaneous blood flow. Conversely, an organism can dissipate heat by reducing thermogenesis, seeking a cooler environment, increasing cutaneous blood flow, and increasing evaporative heat loss through panting and sweating (Tan and Knight, 2018). While many of these processes occur peripherally, they are regulated by the central nervous system.

Regulation of body temperature and metabolism are energetically demanding processes that are controlled by a region of the anterior hypothalamus known as the preoptic area (POA) (Tan and Knight, 2018). The POA has long been associated with thermoregulation, as c-Fos immunoreactivity increases following warm-exposure and activation of neurons within this nucleus induces a behavioral and physiological response commensurate with heat exposure (e.g., panting and decrease in core body temperature) (Scammell et al., 1993; Bachtell et al., 2003; Tan et al., 2016). Most mammalian biological processes must occur within a narrow thermal range. Given the energetic cost of thermogenesis, the POA must keep this process tightly regulated: Inappropriate thermogenesis wastes stored energy, and, in times of decreased food availability, thermogenesis must be limited to conserve energy.

Recent advances have increased our understanding of the central processes that occur within the hypothalamus for endothermic mammals to regulate temperature, metabolism, and torpor/hibernation, many of which converge upon a single neuronal cell type that when activated, is sufficient to drive these behavioral and physiologic responses.

## Numerous Genetic Markers of Thermoregulatory Neurons Are Expressed in a Single Preoptic Cellular Cluster

**FIGURE 1:**
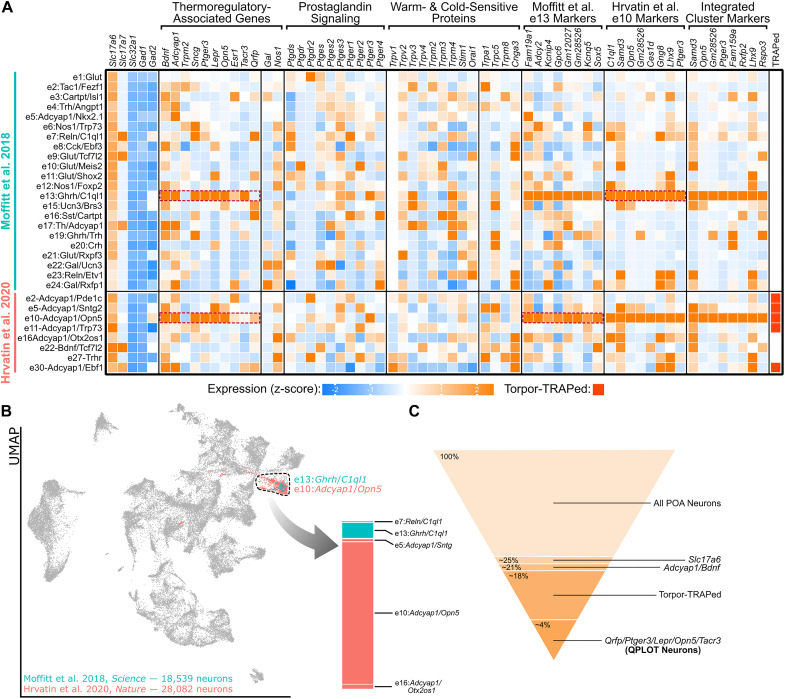
A single molecular identity for QPLOT neurons. **(A)** Heatmap of excitatory POA neurons from Moffitt et al. (2018) and Hrvatin et al. (2020) Known marker genes for thermoregulatory POA neurons, prostaglandin-associated genes, and temperature-sensitive proteins are indicated in columns. Additionally, the top 8 marker genes for excitatory cluster 13 (e13) from Moffitt et al. (2018) and excitatory cluster 10 (e10) from Hrvatin et al. (2020) are provided as well as marker genes for the indicated cluster representing QPLOT neurons when the datasets are merged. Clusters identified in Torpor-TRAP experiments (Hrvatin et al., 2020 clusters only) are indicated. Data is represented as relative expression of each gene within the cluster, averaged over all neuronal clusters, including inhibitory neurons (shown in Supplementary Figure 1). **(B)** Uniform manifold projection of 46,621 POA neurons integrated with Seurat v3 (Stuart et al., 2019). Cluster 16, the cluster containing QPLOT neurons, is indicated by the dashed black line. The QPLOT neuron cluster is composed predominantly of e13 from Moffitt et al. (2018) and e10 from Hrvatin et al. (2020) **(C)** Summary of neuronal populations within the POA that are sufficient to reduce body temperature and thermogenesis (Supplementary Figure 2).

Warm-responsive neurons (WRNs) are neurons that are activated by exposure to warm stimuli, often indicated by increased expression of c-Fos or phosphorylated riboprotein S6 (pS6) (Sheng and Greenberg, 1990; Knight et al., 2012). This activation, in turn, produces a response that decreases body temperature through inhibition of thermogenesis, redistribution of blood flow to superficial tissues, cold-seeking behavior, and decreased activity (Tan and Knight, 2018). Reactivation of neurons that induce c-Fos following warm exposure results in a decrease in body temperature, even when reactivation occurs at room temperature (Harding et al., 2018). A population of WRNs in the POA are known to be excitatory as chemogenetic activation of POA Vglut2^+^ neurons, but not Vgat^+^ neurons, results in a decrease in energy expenditure and core body temperature (Song et al., 2016; Zhao et al., 2017). The identity of WRNs has been further elucidated based on expression of *Adcyap1* and *Bdnf* as optogenetic activation of either neuronal subset decreases core body temperature and facilitates cold-seeking behavior. Furthermore, both *Bdnf* and *Adcyap1* colocalize with pS6 following warm stimulation (Tan et al., 2016). While these markers help to define the molecular identity of WRNs, numerous excitatory POA populations co-express these genes (Figure 1A; Moffitt et al., 2018). Another approach that has been used to identify preoptic cellular populations that regulate energy expenditure is to use a tamoxifen-inducible CreER at the *Fos* locus for targeted recombination in active populations (TRAP). When tamoxifen is administered during fasting-induced torpor (torpor-TRAP), neurons that are active during this state of reduced metabolism and thermogenesis can be used to increase expression of cre-dependent genes, such as a fluorescent reporter or designer receptor exclusively activated by designer drugs (DREADDs). Chemogenetic reactivation of torpor-TRAPed neurons in the POA is sufficient to decrease core body temperature, suggesting that at least one of the torpor-TRAPed cell types is able to regulate thermogenesis (Hrvatin et al., 2020). Sequencing of these torpor-TRAPed neurons identifies numerous populations, several of which are excitatory and express both *Bdnf* and *Adcyap1* (Figure 1A).

Several additional studies, using different genetic and molecular markers, have been able to precisely identify which of the torpor-TRAPed Bdnf^+^/Adcyap1^+^ neuronal populations are responsible for suppressing energy expenditure and decreasing body temperature. Chemogenetic activation of POA neurons using either *Lepr-cre*, *Qrfp-cre*, *Opn5-cre*, or *Esr1-cre* were each individually sufficient to decrease core body temperature and energy expenditure (Yu et al., 2016; Takahashi et al., 2020; Zhang K.X. et al., 2020; Zhang Z. et al., 2020). Additionally, neurokinin B (NKB) signaling within the POA via neurokinin 3 receptor (NK3R; gene: *Tacr3*) results in a rapid drop in body temperature. Furthermore, both *Lepr* and *Sncg* have been colocalized to c-Fos immunoreactivity following warm exposure (Yu et al., 2016; Moffitt et al., 2018). Two independent single-cell/nucleus RNA-sequencing datasets of the POA indicate that these genetic markers of thermoregulatory neurons comprise a single neuronal cluster, identified as e13:*Ghrh/C1ql1* by Moffitt et al. (2018) and e10:*Adcyap1/Opn5* by Hrvatin et al. (2020) (Figure 1A). Between these datasets, markers for these clusters overlap and when neurons from the datasets are integrated, a joint cluster is formed as a superimposition of the e10:*Adcyap1/Opn5* and e13:*Ghrh/C1ql1* clusters (Figures 1A,B), suggesting that both datasets have identified the same neuronal cell type. From this integrated cluster, *Ptger3*, which encodes the fever-mediating prostaglandin EP_3_ receptor (EP_3_) and is expressed in *Qrfp-cre* neurons, and *Opn5* are among the most conserved markers for this population (Lazarus et al., 2007; Takahashi et al., 2020; Figure 1A).

Taken together, these studies demonstrate that *Lepr*-, *Opn5*-, and *Qrfp-*expressing POA neurons comprise the same cellular population and that activation of this excitatory population is sufficient to decrease metabolism and body temperature. Using progressively more specific cell markers, the molecular identity of these WRNs begins to emerge (Figure 1C). A name for this warm-responsive POA population has recently been proposed: quiescence-inducing neurons or Q neurons, based on their ability to induce a hibernation-like state when activated, as well as a play on their expression of the neuropeptide pyroglutamylated RFamide peptide or Qrfp (Takahashi et al., 2020).

It is important to mention several limitations of referring to these neurons as “quiescence-inducing”. First, these neurons function in more than promoting quiescence, including in temperature and metabolic regulation. Second, not all species undergo similar quiescent states, whether ranging from hibernation to torpor or deep sleep. While these cells may be sufficient to induce quiescence in species that are capable of entering such a state, it is likely that this cellular population exists in all endotherms to regulate energy expenditure. Lastly, *Qrfp* is just one marker among many that is useful in molecularly defining this population. Thus, we propose an expanded definition of the previously defined Q neuron and suggest the name QPLOT neurons, for the markers *Qrfp*, *Ptger3*, *Lepr*, *Opn5*, and *Tacr3* that identify this population. The use of two or more of these markers will allow for precise identification of the population of neurons.

## QPLOT Neurons as Central Integrators of Temperature and Metabolic Information

**FIGURE 2:**
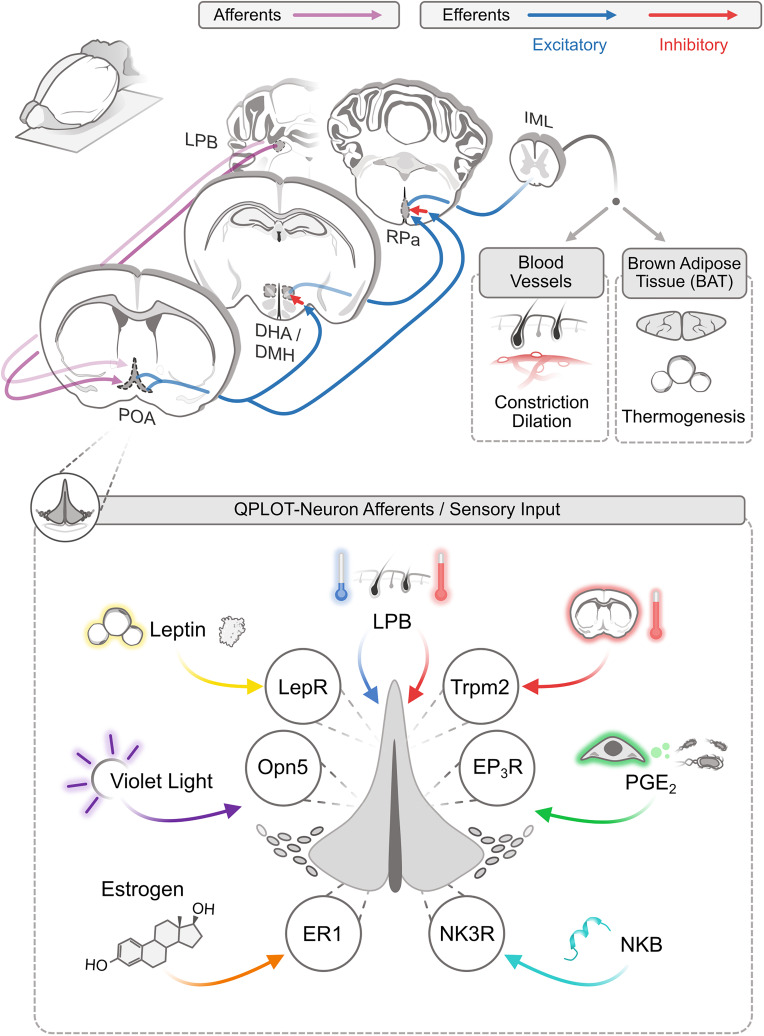
Afferent and efferent thermoregulatory signaling for QPLOT neurons. Schematic of connectivity of QPLOT neurons that mediate body temperature regulation. QPLOT neurons are responsive to numerous stimuli that are integrated to produce the appropriate response.

Given the data suggesting that these previous studies have been investigating a singular neuronal population, their published data can be reanalyzed to better understand some of the characteristics of QPLOT neurons. As a unified cellular population, QPLOT neurons can integrate a variety of stimuli to regulate thermogenesis and metabolism. These stimuli include ascending signals from warm and cold sensors in the skin, local changes in hypothalamic temperature, inflammatory-mediated prostaglandin signaling, the hormone leptin, violet light, and estrogen (Figure 2).

### Ascending Temperature Information

Ambient temperature is detected by cutaneous warm-sensitive and cold-sensitive sensory neurons, which predominantly utilize TRP channels to detect the external temperatures. Trpm8 is necessary for cold-sensitive sensory neurons to detect diminished temperature, whereas Trpv1 is the predominant warm-sensitive protein expressed in sensory neurons (Knowlton et al., 2010, 2013; Pogorzala et al., 2013; Yarmolinsky et al., 2016). This temperature information is conveyed to the brain through multiple pathways, including projections to the lateral parabrachial nucleus (LPB), which in turn projects to the POA. Prior to the POA, warm and cold information is transmitted independently (Geerling et al., 2016). Within the POA, these temperature cues converge, such that activity can be modulated by external increases or decreases in temperature (Morrison and Nakamura, 2011; Yang et al., 2020). Consistent with this model, the LPB has been monosynaptically traced from POA neurons expressing *Opn5*-cre (Zhang K.X. et al., 2020). These results would indicate that QPLOT neurons themselves receive at least one of these temperature streams directly, rather than through a local circuit within the POA. Thus, in response to ascending external cold or warm stimuli, QPLOT neuron activity can be modulated to adjust to external temperatures or alternatively, interpret external temperature as a cue, along with other stimuli, that the organism should enter torpor.

### Intrinsic Temperature Sensitivity

In addition to receiving peripheral thermal information, neurons of the POA have long been known to be intrinsically temperature-sensitive with increased firing rate in response to local increases in temperature and decreased firing in response to local temperature decreases (Magoun et al., 1938; Nakayama et al., 1961, 1963). There is an additional POA neuronal population that functions conversely to these intrinsically warm-sensitive neurons (iWSNs), in which increasing the temperature decreases firing rate while decreasing the hypothalamic temperature increases firing. However, this “cold response” is dependent on synaptic neurotransmission, suggesting that these cells lack an intrinsic ability to respond to local changes in temperature, but are rather responding to extrinsic activity, possibly in direct response to the iWSNs (Kelso and Boulant, 1982; Boulant, 2006; Wang et al., 2019). These iWSNs and cold-responsive POA neurons (CRNs) represent two distinct neuronal populations that likely function together to regulate energy expenditure and the response to external and internal thermal stimuli (Wang et al., 2019).

The molecular identity of iWSNs has not been clearly identified, however, the temperature-sensitive cation channel, Trpm2, is necessary for warm-sensitivity within the POA and functions to limit the fever response induced by either PGE2 or both IL1β and IL6 (Song et al., 2016). *Trpm2* is found in *Opn5*-expressing neurons and chemogenetic activation of *Trpm2*-cre mice results in a decrease in body temperature, demonstrating that Trpm2 is expressed in a neuronal population sufficient to decrease body temperature and metabolism, likely conferring the cell intrinsic temperature sensitivity (Song et al., 2016; Zhang K.X. et al., 2020). Unfortunately, Trpm2 is expressed in a variety of excitatory and inhibitory POA neurons cell types and, therefore, does not serve as a useful marker of any individual population (Song et al., 2016). Thus, Trpm2 is necessary for intrinsic temperature sensitivity of the POA, particularly at high temperatures, but many neuron types within the POA beyond QPLOT neurons utilize this cation channel. There may still be additional temperature-sensitive proteins within the POA that function closer to homeostatic temperatures, rather than at pyretic temperatures. An assumption that has likely limited the molecular identification of iWSNs is that multiple neuronal cell types may be intrinsically temperature sensitive within the POA and that QPLOT neurons only represent one of these. For example, WRNs may use temperature-sensitive proteins, but so could POA neurons involved in the regulation of thirst or sleep. Available sequencing datasets are consistent with this hypothesis, in which no temperature-sensitive proteins are specific to a single neuronal cluster (Figure 1A).

### Prostaglandin Signaling

*Ptger3* is one of the most selective marker genes for QPLOT neurons (Figure 1A) and is likely integral to their physiology. Prostaglandins are produced by endothelial cells and microglia within the brain following exposure to endotoxins or inflammatory cytokines (Eskilsson et al., 2017). Prostaglandin EP3 receptor (EP3), encoded by *Ptger3*, is activated by PGE2 to mediate the fever response (Lazarus et al., 2007). This pathway can be suppressed by COX inhibitors to block the synthesis of PGE2, thus reducing fevers. Additionally, targeted ablation of excitatory neurons in the POA or deletion of *Ptger3* from the hypothalamus are sufficient to abolish the inflammatory-induced fever response (Lazarus et al., 2007; Machado et al., 2020). It is clear that both approaches would disrupt the QPLOT neuron population. Accordingly, EP3 activation (G_*i*_-coupled receptor) should reduce QPLOT neuron activity and thus, increase body temperature. In hypothalamic slices, PGE2 has been shown to decrease the firing rate of warm-sensitive neurons, while increasing the activity of temperature-insensitive neurons (Ranels and Griffin, 2003). These results are consistent with a model in which QPLOT neurons are intrinsically temperature-sensitive, express EP3, and upon PGE2 stimulation, decrease their activity, resulting in an increase in body temperature. However, this PGE2-EP3 mechanism may be limited to the inflammatory response, as local changes in hypothalamic temperature do not appear to change PGE2 concentration (Wang et al., 2019).

### Leptin Signaling

Leptin is a white adipocyte-derived hormone that conveys information on nutritional status (proportional to adipocyte mass) to other regions of the body, primarily to several hypothalamic brain regions, including the arcuate nucleus (ARC), dorsomedial hypothalamus (DMH), and POA (Schwartz et al., 1996). Systemic leptin regulates food intake and body temperature to correct energy balance during fed and fasting states (Ahima et al., 2000), however these functions are predominantly mediated via leptin receptor (Lepr) activity in the ARC and DMH, respectively (Cowley et al., 2001; Balthasar et al., 2004; Gropp et al., 2005; Luquet et al., 2005; Enriori et al., 2011; Fischer et al., 2016). Activation of *Lepr*-expressing POA neurons decreases food intake, thermogenesis, and energy expenditure, even at thermoneutrality, but local application of leptin into the POA does not alter energy expenditure and only modestly reduces acute food intake (Yu et al., 2016, 2018). Counterintuitively, local deletion of the leptin receptor does not change food intake or energy expenditure when fed; however, a difference in energy expenditure emerges when mice are fasted or given a high fat diet (Yu et al., 2018). Thus, most of the central functions ascribed to leptin can be sufficiently explained via *Lepr*-expressing neurons in the DMH and ARC, though roles for leptin signaling within the POA are emerging.

Interestingly, in the proposed QPLOT neuron, Lepr is functionally positioned to explain several observations. First, following food restriction at subthermoneutral temperatures (a state that would normally induce torpor), leptin treatment prevents decreases in body temperature (Fischer et al., 2016). Second, mice deficient in leptin spontaneously enter torpor in the absence of fasting or cold exposure (Swoap, 2008). Third, the PGE2-induced fever response is attenuated in fasted mice (Inoue et al., 2008). Since fasting is a state that results in a marked decline in leptin, QPLOT neurons may respond to this drop in hormone level by modulating the thermogenic response to other stimuli. In this manner, the internal metabolic state could directly influence energy utilization when thermoregulation must otherwise be upregulated via integration through a single cellular population. In these examples, the response to the lack of leptin during times of fasting may be the interpreted signal, rather than to increases in leptin.

### Violet Light and Opn5

*Opn5* encodes the violet light-sensitive atypical opsin, neuropsin, and serves as one of the most specific markers for QPLOT neurons (Figure 1A). In birds, neuropsin is known to be expressed in hypothalamic tanycytes, where it regulates seasonal physiology and behavior, although it is not detected in mammalian tanycytes (Tarttelin et al., 2003; Nakane et al., 2010; Yoo et al., 2019). Stimulation of the POA with violet light activates *Opn5-cre* labeled neurons and results in a suppression of metabolism and thermogenesis. This response is absent from *Opn5*-deficient mice. In the absence of *Opn5*, mice have an elevated metabolic rate and body temperature, similar to effects seen following inhibition of putative QPLOT neurons (Song et al., 2016; Zhang K.X. et al., 2020). Despite expression in different cell types between birds and mammals, the functions of hypothalamic *Opn5* in migration, reproduction, metabolism, and torpor suggest a conserved role for Opn5 in regulating seasonal physiology.

### Estrogen Receptor

Through a variety of peripheral and central mechanisms, estrogen promotes heat loss and reduced thermogenesis. Estrogen receptor alpha (ERα, *Esr1* gene) is expressed in several POA neuronal populations, including in the QPLOT cluster (Figure 1A). Sensitivity to both temperature and estrogen has been noted in a proportion of POA neurons, consistent with *Esr1*-expression in temperature-sensitive QPLOT neurons (Silva and Boulant, 1986; Zhang Z. et al., 2020). Additionally, chemogenetic activation of *Esr1*-cre neurons of the POA leads to a suppression of body temperature and metabolism (Zhang Z. et al., 2020). γ-synuclein, encoded by the warm-induced gene *Sncg*, is known to modulate estrogen signaling, indicating that signaling through ERα may be differentially modulated according to body temperature (Jiang et al., 2004; Moffitt et al., 2018). Taken together, QPLOT neurons appear to be sensitive to estrogen, although how this highly dynamic signaling integrates with other stimuli at the level of the POA remains unclear.

### Neurokinin B and NK3R

Kisspeptin-Neurokinin B-Dynorphin-expressing (KNDy) neurons of the arcuate nucleus are another population of estrogen-sensitive neurons that function, in part, in thermoregulation (Rance et al., 2013). These neurons are believed to function in hot flashes. Activation of KNDy projections to the preoptic are sufficient to evoke superficial vasodilation, a decrease in core body temperature, and induction of c-Fos in the POA (Padilla et al., 2018). The thermoregulatory response to KNDy neuron activation is blocked via preoptic inhibition of neurokinin signaling. Since *Tacr3* encode NK3R, the receptor for NKB, and *Tacr3* is enriched in the QPLOT neuronal cluster, it is likely that activation of QPLOT neurons is necessary for KNDy neuron-mediated vasodilation and suppression of body temperature.

## Activation of QPLOT Neurons and Their Efferent Targets

The main thermoregulatory efferents of POA neurons project to the DMH and raphe pallidus (RPa). These projections are excitatory although the targets of these neurons are believed to be inhibitory, a view that has changed in recent years (Saper and Machado, 2020). Activation of QPLOT neurons through various cre lines, either optogenetic or chemogenetic, results in a decrease in body temperature and energy expenditure (Song et al., 2016; Tan et al., 2016; Yu et al., 2016; Hrvatin et al., 2020; Takahashi et al., 2020; Zhang K.X. et al., 2020; Zhang Z. et al., 2020). This effect is likely due to a combination of cutaneous vasodilation, reduced food intake, and a suppression of non-shivering thermogenesis (Abbott and Saper, 2017). Projections from the POA to the DMH convey thermogenic information, as optogenetic activation of *Qrfp-cre* projections to the DMH is sufficient to drive a sustained suppression of BAT thermogenesis. By contrast, activation of *Qrfp-cre* projections directly to the RPa has only a transient suppression of BAT thermogenesis (Takahashi et al., 2020). Similarly, inhibition of the DMH attenuates PGE2 evoked thermogenesis but has no effect on cutaneous vasoconstriction (Rathner et al., 2008). Inhibition of the RPa also inhibits PGE2 evoked thermogenesis, although this could be due to inhibition of direct POA-RPa projections or the POA-DMH-RPa pathway (Yoshida et al., 2009; Zhang et al., 2011). While these two parallel pathways result in similar effects on body temperature, individual neurons in the POA largely project to only one of these efferents, as simultaneous retrograde tracing from the DMH and RPa identifies very few co-labeled neurons (Nakamura et al., 2009). Thus, a subset of QPLOT neurons likely project to the DMH, to regulate thermogenesis, while another subset of QPLOT neurons may project directly to the RPa to regulate vasodilation and potentially additional regulation of thermogenesis. Despite evidence of at least two anatomically distinct populations, there is currently only evidence for one molecularly-defined cellular population. Whether these differences in projection arise from cellular identity/fate decisions or are a result of developmental cues regulating axon guidance remains to be determined. Both the direct and indirect projections reconvene at the RPa, where descending neurons of the RPa project to the preganglionic sympathetic neurons of the IML (intermediolateral cell column) (Nakamura et al., 2004). From the IML, sympathetic neurons innervate BAT and cutaneous blood vessels (Figure 2). In addition to these targets of QPLOT neurons, they also project to the PVN, VMH, and ARC where they likely exert additional influence. These informative studies will need to be revisited with specific markers of the QPLOT neuron population to validate and functionally expand this metabolic circuitry.

A separate thermoregulatory population in the ventrolateral preoptic area (VLPO) is an inhibitory galanin (Gal)-expressing neuron that when activated, promotes NREM sleep and a decrease in core body temperature and when ablated, results in a higher baseline body temperature (Kroeger et al., 2018; Ma et al., 2019). *Gal* is noticeably absent from the QPLOT cluster, although it is expressed in several other clusters (Figure 1A; Supplementary Figure 1; Guo et al., 2020). Interestingly, VLPO neurons inhibit shivering induced by PGE2 infused into the POA and additionally can inhibit RPa-induced thermogenesis (Conceição et al., 2018). It is conceivable that this VLPO^*Gal*^ population converges with QPLOT neuron efferents to modulate body temperature and energy expenditure (Zhao et al., 2017; Conceição et al., 2018). Alternatively, or additionally, there may be a local circuit within the anterior hypothalamus between these two thermoregulatory populations. Consistent with this hypothesis, viral tracing from *Qrfp-cre* neurons indicates connectivity within the VLPO and axons from the POA form excitatory synapses with Gal-expressing neurons in the VLPO (Walter et al., 2019; Takahashi et al., 2020). More work with precise genetic markers will be needed to further understand how these separate neuronal populations may function together to regulate body temperature and arousal.

## Conclusion

Currently available single cell/nucleus sequencing datasets of the POA suggest that there is a unified cellular population known as QPLOT neurons that express *Qrfp*, *Ptger3*, *Lepr*, *Opn5*, *Tacr3*, *Trpm2*, *Esr1*, *Sncg*, *Adcyap1*, and *Bdnf*, that when activated, results in a decrease in core body temperature and energy expenditure. QPLOT neurons are a key cellular population that regulate energy expenditure and thermogenesis by integrating ascending temperature stimuli, local hypothalamic temperature, the internal metabolic state via adipocyte-derived leptin, inflammatory-mediated prostaglandin signaling, ambient violet light, estrogen, and neurokinin signaling. Together, these stimuli modify the activity of QPLOT neurons which function with other neurons in a thermoregulatory circuit to appropriately balance energy utilization and body temperature, keeping an organism within a tightly regulated physiologic range.

## Data Availability Statement

The datasets analyzed for this study can be found in the Gene Expression Omnibus (GSE113576 and GSE149344); https://www.ncbi.nlm.nih.gov/geo/query/acc.cgi?acc=GSE113576; https://www.ncbi.nlm.nih.gov/geo/query/acc.cgi?acc=GSE149344.

## Author Contributions

RL and BU conceived the manuscript. BU drafted and revised the manuscript in discussion with RL and SD’S. BU composed Figure 1. SD’S composed Figure 2. All authors approved of the submitted version.

## Conflict of Interest

The Lang lab has a sponsored research agreement with BIOS Lighting Inc. The authors declare that the research was conducted in the absence of any commercial or financial relationships that could be construed as a potential conflict of interest.

## Supplementary Material

Supplementary Figure 1 Heatmaps of excitatory, inhibitory, and hybrid POA neurons from **(A)** Moffitt et al. (2018) and **(B)** Hrvatin et al. (2020) Known marker genes for thermoregulatory POA neurons, prostaglandin-associated genes, and temperature-sensitive proteins are indicated in columns. Additionally, the top 8 marker genes for excitatory cluster 13 (e13) from Moffitt et al. (2018) and excitatory cluster 10 (e10) from Hrvatin et al. (2020) are provided as well as marker genes for the indicated cluster representing QPLOT neurons when the datasets are merged. Clusters identified in Torpor-TRAP experiments (Hrvatin et al., 2020 clusters only) are indicated. Data is represented as relative expression of each gene within the cluster, averaged over all neuronal clusters.

Supplementary Figure 2 Uniform manifold approximation and projection of integrated single cell/single nucleus RNA-sequencing datasets. Moffitt et al. (2018) and Hrvatin et al. (2020) datasets were integrated in Seurat v3 using 3,000 integration features. UMAP with cells colored based on **(A)** dataset of origin, **(B)** original classification as excitatory, inhibitory, or cholinergic, and **(C)** integrated cluster ID using a resolution of 2.0.

Supplementary Table 1 QPLOT Neuron Conserved Markers. Marker genes for the QPLOT neuron cluster from integrated POA single cell/nucleus sequencing datasets.
