# Supplementary figures and images for: QPLOT Neurons—Converging on a Thermoregulatory Preoptic Neuronal Population
Source: Front Neurosci. 2021 May 4;15:665762. doi: 10.3389/fnins.2021.665762 (PMC8130930; doi:10.3389/fnins.2021.665762)

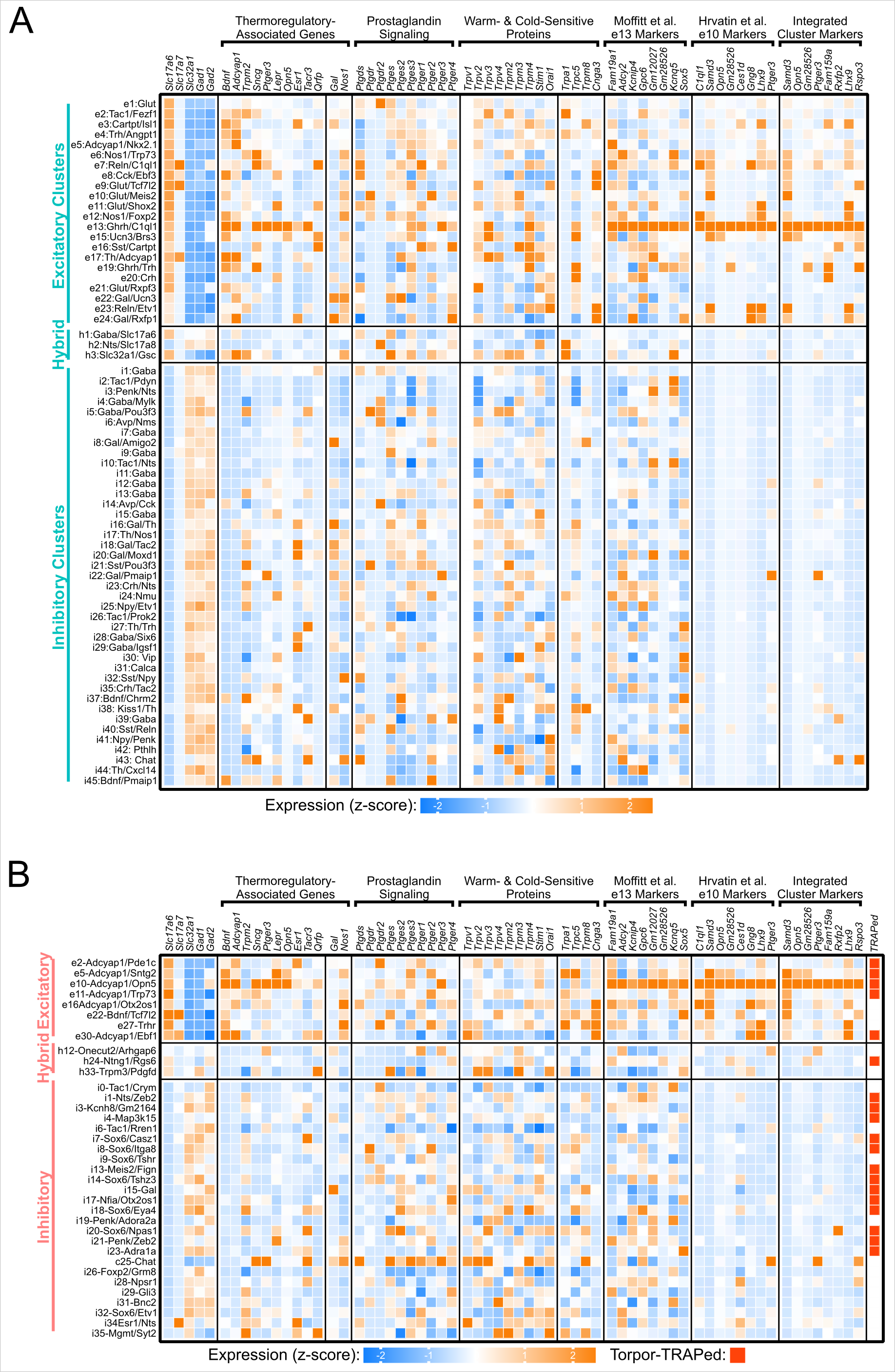

Supplement: Supplementary Figure 1 — Heatmaps of excitatory, inhibitory, and hybrid POA neurons from (A) Moffitt et al. (2018) and (B) Hrvatin et al. (2020) Known marker genes for thermoregulatory POA neurons, prostaglandin-associated genes, and temperature-sensitive proteins are indicated in columns. Additionally, the top 8 marker genes for excitatory cluster 13 (e13) from Moffitt et al. (2018) and excitatory cluster 10 (e10) from Hrvatin et al. (2020) are provided as well as marker genes for the indicated cluster representing QPLOT neurons when the datasets are merged. Clusters identified in Torpor-TRAP experiments (Hrvatin et al., 2020 clusters only) are indicated. Data is represented as relative expression of each gene within the cluster, averaged over all neuronal clusters. [file Image_1.TIFF]

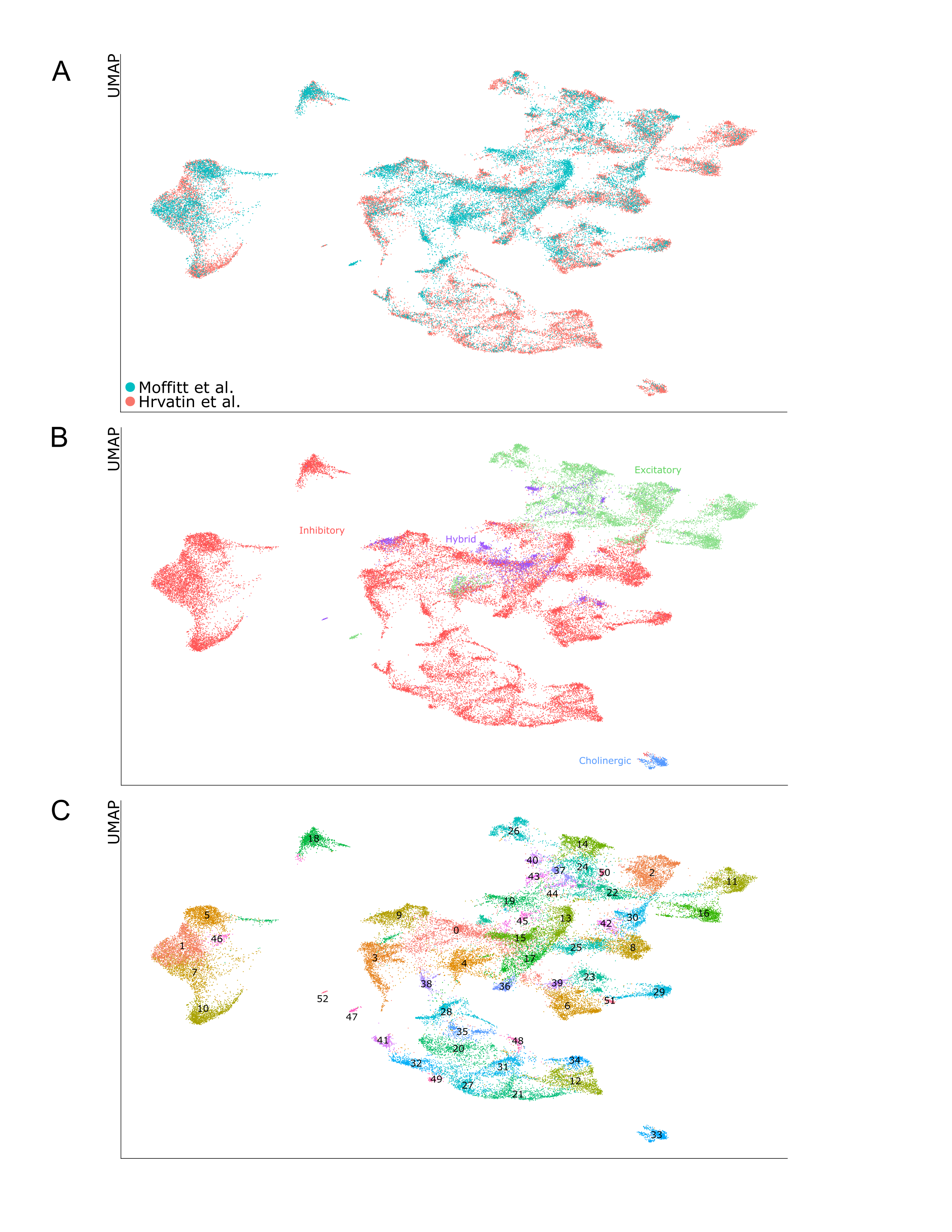

Supplement: Supplementary Figure 2 — Uniform manifold approximation and projection of integrated single cell/single nucleus RNA-sequencing datasets. Moffitt et al. (2018) and Hrvatin et al. (2020) datasets were integrated in Seurat v3 using 3,000 integration features. UMAP with cells colored based on (A) dataset of origin, (B) original classification as excitatory, inhibitory, or cholinergic, and (C) integrated cluster ID using a resolution of 2.0. [file Image_2.png]
